# Supplementary material for: Botanical, Phytochemical, Anti-Microbial and Pharmaceutical Characteristics of Hawthorn (Crataegus monogyna Jacq.), Rosaceae
Source: Molecules. 2021 Nov 30;26(23):7266. doi: 10.3390/molecules26237266 (PMC8659235; doi:10.3390/molecules26237266)
Supplement: Supplementary file 1 [file molecules-26-07266-s001.zip › molecules-1388126 supp final.pdf]

Supplementary Table S1. Molecular techniques used for genetic characterization of *C. monogyna* and related references.

Technique	References
RAPDs	Ferrazzini et al., 2008
	Yilmaz et al., 2010
	Rajeb et al., 2010
	Serçe et al., 2011
SSRs	Khiari et al., 2015
	Güney et al., 2018

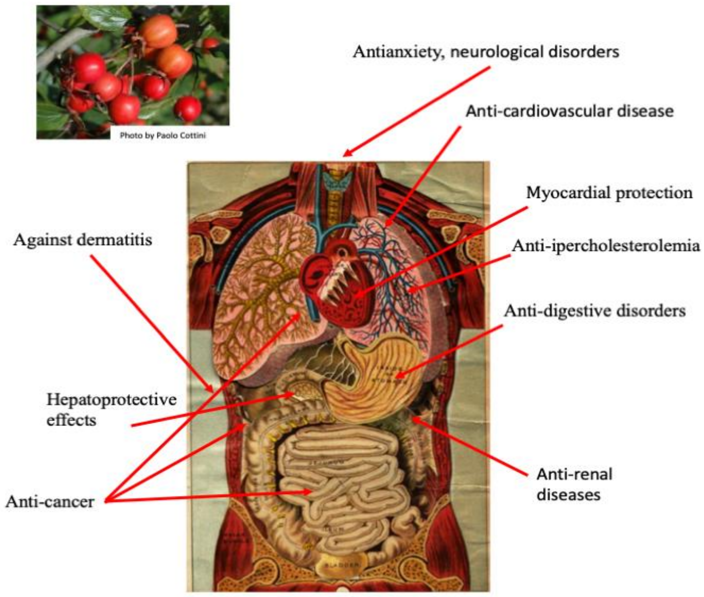

Supplementary Figure S1. Pharmaceutical action of *C. monogyna* on degenerative and chronic diseases of different human organs.
